# Quacks In and Out of the Profession

**Published:** 1890-09

**Authors:** 


### EDITORIAL DEPARTMENT.

F. E. DANIEL, M. D., EDITOR AND PUBLISHER.

THIS JOURNAL, by unanimous vote of the members, was made the Official Organ of the Austin District Medical Society.

COLLABORATORS:

*Dr. R. M. Swearingen, Austin.*
*Prof. B. E. Hadra, M. D., Galveston.*
*Prof. Geo. Cupples, M. D., San Antonio.*
*Dr. T. C. Osborn, Cleburne, Texas.*
*Dr E. J. Doering, Chicago.*
*Dr. E. J. Beall, Fort Worth.*
*Dr Odo Betz, Germany.*
*Prof. W. B. Rogers, M. D., Memphis.*
*Dr. J. W. McLaughlin, Austin.*
*Dr. T. J. Tyner, Austin.*
*Prof. J. F. Y. Paine, M. D., Galveston.*
*Dr. R. H. L. Bibb, Mexico.*
*Dr. T. J. Bennett, Austin.*
*Dr. Bat Smith, Wharton.*
*Dr. E. Meierhof, New York.*
*L. H. Luce, M. D., West Tisbury, Mass.*

### QUACKS IN AND OUT OF THE PROFESSION.

### Sauce for the Gander.

That Texas should be overrun with quacks and pretenders, men who are totally unqualified by education to practice medicine, would naturally be supposed from the laxity of our laws,—or, rather, because there is no law in Texas which requires that a man shall know anything of medicine in order to practice it. This, together with stringent laws in other States acting as a *vis a tergo*, has filled Texas with illegitimate Doctors. They naturally drift Texas-ward; and finding here a "suitable nidus," as they say of other parasites, flourish like a green bay tree; there is none to molest them nor make them afraid.

But are these the only quacks in Texas, the unlettered and undiplomaed? By no means. It has become the custom to regard as a quack every man practicing without a diploma (or a license from an examining board,) as well as disciples of eclecticism, etc.; in brief, to regard a diploma as *the* distinguishing feature between the "regular" and the "irregular."

Webster defines a quack to be "one who boasts of knowledge not possessed; an ignorant pretender." By this standard,—

and it is the highest authority,—can it not be shown that there are quacks in the regular medical ranks? aye, in the very *sanctum sanctorum* of the Texas State Medical Association? and hence, that all attempts to "suppress quackery" must necessarily prove futile? We may and should have a law which would restrict the right to practice medicine to those who can show to a legal authority that by education and training they are competent to do so; but such a law could not be made retro-active, and could not therefore reach and remedy the existing evil; if properly administered, it could only prevent further mischief to a limited extent.

There is a type of the genus quack as generally understood by the profession, yet, one who, judged by Webster's definition is far from being a quack—strikingly illustrated in the person of a splendid scamp who recently flourished at New Braunfels. By those who know him he is said to be a man of fine attainments, well informed on all subjects germane to medicine and surgery, and practices both, *secundem artem*. But he is guilty of having *no diploma*, and therefore, of not fraternizing with the regular profession, of snapping his fingers at the code; in short, of ignoring all professional amenity. He devotes himself solely to money-getting and pleasure, drives four-in-hand,—high-steppers,—wears purple and fine linen, and fares sumptuously every day. He is equipped with the latest and best books, and has the finest surgical armamentarium extant. But everything is directed to the one end,—to getting money. Like Tigg Montague of "Anglo-Bengalee" fame, even his sumptuous dinners, where "wine is like water," are made subservient to it; he knows human nature, and assails its weakness by every device, and so that he gets money, has no regard for consequences. He is said to have carried away $30,000 from the rural village of New Braunfels, those primitive, credulous and impressive burghers—descendants of the plain people who followed Baron Braunsfels to those sylvan streams. He is now in San Antonio we learn, "cutting a wide swath," and still "coining money." Is *he* a quack? If so how shall *he* be suppressed?

But all the quacks are not of the illiterate sort, nor of the undiplomaed sort; nor yet of the Von Kenner sort. Some have genuine diplomas, and are high up in the social and professional current, swimming along smoothly till may be, some unlucky chance,—some *libel suit*, perhaps,—may bring to light their ignorance, if not their guilt. Some even essay to take the lead, and jealous of the State Association's welfare, constitute themselves its guardian, making the preservation of harmony their bounden duty; "harmony," at whatever cost! How can these quacks be suppressed? Who shall watch the watchman?

And it strikes us that these are the worst and most dangerous of the genus quack. Protected by a diploma and screened in the bosom of the State Association, they are safe, or nearly safe from detection. Their ignorance, their mistakes in diagnosis and treatment seldom come to light, and the deaths resulting therefrom can, with comparative safety be "laid to the Lord," the refuge of all such.

Were a man having no diploma, *ergo*, a quack (?)—to have a young woman of 20 in the service of his family, and she should let her foot slip and become pregnant; were he to see her every day, in fact, have her consult him about *suppressed menses*; to observe the languor and loss of appetite, the morning sickness and salivation of early pregnancy; later, note the change in her figure and the gradual enlargement of the breast and of the abdomen; in brief, to have her under his eye, and actual professional care, during the whole period of gestation from fecundation to the verge of actual labor, and never, never once suspect pregnancy, nor propose an examination, no, not even when his wife, with her woman's intuition suggests "Doctor, 'aint Sairey big?" "Quack" would be too soft a name for this fellow; but suppcse that all the foregoing should occur in the experience of a noted quack, one who had been making big pretensions as a gynecologist, and "considerable if not more" pretensions as an obstetrician and accoucheur,—would he not be the laughing stock of the regular profession? Would he not be an "ignorant pretender?" Aye, would not every so-called "journal" in the land having a vocabulary of its own, a vernacular, the vulgarity of which for strength and originality would shame billingsgate or put to blush the fishmongers of New Market, find such a man a target for its choicest and most vulgar epithets? and in its nastiest mood, seek in vain to do the subject justice? Aye, a law to suppress quackery and a State board of health would be loudly demanded, right off, no delay. "Quackery must be suppressed."

But our Christian friends and fellow sufferers, what if such a thing were to actually occur in the experience of one of the elect? One who has filled Presidential chairs, who makes speeches at banquets, and writes articles for the obstetric journals? Would *he* not be an "ignorant pretended?" Come, now; "sauce for the goose," you know, brethren, *ought* to be "sauce for the gander;" and a quack is a quack, no matter how luckily he may have evaded a detection all these years. Ought not such total ignorance of all the signs of pregnancy to relegate him to the ranks of "ignorant pretenders," and stamp him as a quack? What though he have a deploma? What though he may possibly be an experienced legislator, and know precisely what the profession needs in the way of a law to suppress quackery, (that a disgusted constituency may have hissed him on his return is not to the purpose;)—what though he may have political influence, especially with the Republican party, shall that screen him? Will it bring to life again any who may have been the victims of his ignorance any more than those of other quacks?

Or take that ambitious individual mentioned in Dr. Leake's paper before the Association, (he was a diplomaed gent,) who performed laparotomy for ovarian disease, and removing the ovaries exclaimed, "behold! just as I diagnosed! "cystic degeneration;" the "cystic degeneration" consisting of graaffian vesicles in a state of evolution, in accordance with their destiny and function! Or the one who, to relieve impaction in a shoulder presentation, disarticulated at the shoulder joint, the child being subsequently delivered by turning—in the hands of another man. Are not these diplomaed gents quacks of the most dangerous kind?

In no other profession or calling on earth, does such a state of things exist. Poor encouragement to a man entering upon the study of medicine, to know and to realize that though he devote the best years of his life, and his time, money and best energies to the acquisition of knowledge that will fit him for the performance of the sacred and responsible duties of the physician, the surgeon, he will surely be met in life by those who have spent neither time nor money, nor endeavor, in the pursuit of knowledge, and who really have none; and in the struggle for existence will be distanced by these fellows, or by those who, having acquired a smattering, only, boast and blow themselves into prominence and practice, and perhaps, (the lightest trash sometimes comes to the top, as in potatoes for instance, the small ones get there,) worm themselves into prominence in the State organizations, distancing the student and the meritorious, but modest practitioner, and—in the matter of money-getting from the profession—he will be sadly left. It really does look sometimes as if in this world there is a premium on iniquity. But, these latter fellows, the pretenders, are found out sooner or later; and like water, they gravitate to their proper level in due time. There is now going on in the profession in Texas a "shaking up of dry bones," a sifting process, and the fittest only, in the course of time, will survive.

